# Supplementary material for: Causal Relationship between Aging and Anorexia Nervosa: A White-Matter-Microstructure-Mediated Mendelian Randomization Analysis
Source: Biomedicines. 2024 Aug 16;12(8):1874. doi: 10.3390/biomedicines12081874 (PMC11351342; doi:10.3390/biomedicines12081874)
Supplement: Supplementary file 1 [file biomedicines-12-01874-s001.zip › Supplementary Figure/Figure S4/Singe SNP plot of MR analysis for TL to ubm-b-1892.pdf]

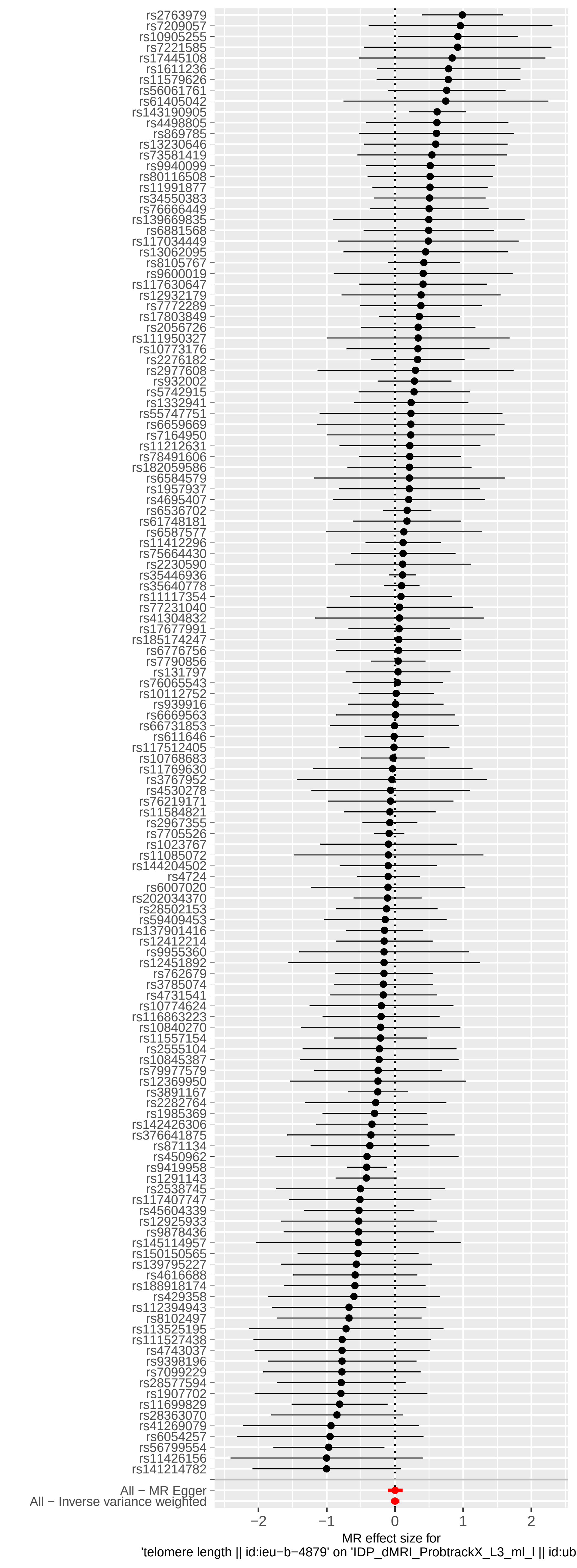
rs2763979
rs7209057
rs10905255
rs7221585
rs17445108
rs1611236
rs11579626
rs56061761
rs61405042
rs143190905
rs4498805
rs869785
rs13230646
rs73581419
rs9940099
rs80116508
rs11991877
rs34550383
rs76666449
rs139669835
rs6881568
rs117034449
rs13062095
rs8105767
rs9600019
rs117630647
rs12932179
rs7772289
rs17803849
rs2056726
rs111950327
rs10773176
rs2276182
rs2977608
rs932002
rs5742915
rs1332941
rs55747751
rs6659669
rs7164950
rs11212631
rs78491606
rs182059586
rs6584579
rs1957937
rs4695407
rs6536702
rs61748181
rs6587577
rs11412296
rs75664430
rs2230590
rs35446936
rs35640778
rs11117354
rs77231040
rs41304832
rs17677991
rs185174247
rs6776756
rs7790856
rs131797
rs76065543
rs10112752
rs939916
rs6669563
rs66731853
rs611646
rs117512405
rs10768683
rs11769630
rs3767952
rs4530278
rs76219171
rs11584821
rs2967355
rs7705526
rs1023767
rs11085072
rs144204502
rs4724
rs6007020
rs202034370
rs28502153
rs59409453
rs137901416
rs12412214
rs9955360
rs12451892
rs762679
rs3785074
rs4731541
rs10774624
rs116863223
rs10840270
rs11557154
rs2555104
rs10845387
rs79977579
rs12369950
rs3891167
rs2282764
rs1985369
rs142426306
rs376641875
rs871134
rs450962
rs9419958
rs1291143
rs2538745
rs117407747
rs45604339
rs12925933
rs9878436
rs145114957
rs150150565
rs139795227
rs4616688
rs188918174
rs429358
rs112394943
rs8102497
rs113525195
rs111527438
rs4743037
rs9398196
rs7099229
rs28577594
rs1907702
rs11699829
rs28363070
rs41269079
rs6054257
rs56799554
rs11426156
rs141214782
All − MR Egger
All − Inverse variance weighted
−2
−1
0
1
2
MR effect size for
'telomere length || id:ieu−b−4879' on 'IDP_dMRI_ProbtrackX_L3_ml_l || id:ub
